# Cholesterol-Independent Effects of Methyl-β-Cyclodextrin on Chemical Synapses

**DOI:** 10.1371/journal.pone.0036395

**Published:** 2012-05-08

**Authors:** Kiel G. Ormerod, Tatiana P. Rogasevskaia, Jens R. Coorssen, A. Joffre Mercier

**Affiliations:** 1 Department of Biological Sciences, Brock University, St. Catharines, Ontario, Canada; 2 Department of Physiology and Biophysics, Faculty of Medicine, University of Calgary, Calgary, Alberta, Canada; 3 Department of Molecular Physiology, School of Medicine and the Molecular Medicine Research Group, University of Western Sydney, Penrith South DC, New South Wales, Australia; University of Waterloo, Canada

## Abstract

The cholesterol chelating agent, methyl-β-cyclodextrin (MβCD), alters synaptic function in many systems. At crayfish neuromuscular junctions, MβCD is reported to reduce excitatory junctional potentials (EJPs) by impairing impulse propagation to synaptic terminals, and to have no postsynaptic effects. We examined the degree to which physiological effects of MβCD correlate with its ability to reduce cholesterol, and used thermal acclimatization as an alternative method to modify cholesterol levels. MβCD impaired impulse propagation and decreased EJP amplitude by 40% (P<0.05) in preparations from crayfish acclimatized to 14°C but not from those acclimatized to 21°C. The reduction in EJP amplitude in the cold-acclimatized group was associated with a 49% reduction in quantal content (P<0.05). MβCD had no effect on input resistance in muscle fibers but decreased sensitivity to the neurotransmitter L-glutamate in both warm- and cold-acclimatized groups. This effect was less pronounced and reversible in the warm-acclimatized group (90% reduction in cold, P<0.05; 50% reduction in warm, P<0.05). MβCD reduced cholesterol in isolated nerve and muscle from cold- and warm-acclimatized groups by comparable amounts (nerve: 29% cold, 25% warm; muscle: 20% cold, 18% warm; P<0.05). This effect was reversed by cholesterol loading, but only in the warm-acclimatized group. Thus, effects of MβCD on glutamate-sensitivity correlated with its ability to reduce cholesterol, but effects on impulse propagation and resulting EJP amplitude did not. Our results indicate that MβCD can affect both presynaptic and postsynaptic properties, and that some effects of MβCD are unrelated to cholesterol chelation.

## Introduction

Cholesterol is an intriguing molecule linked to numerous fundamental physiological functions; thus, alterations in cholesterol metabolism are associated with a range of disorders that include significant current and future healthcare burdens [1], [2]. At least one reason for this extensive linkage of cholesterol to physiological states is that, in contrast to other lipidic membrane components, there are a number of reasonably selective pharmacological tools available with which to modulate the amount and/or effects of cholesterol in cellular membranes [3], [4]. Indeed, this ability to target native cholesterol with reasonable selectivity, coupled with the availability of sensitive quantitative assays, has led to cholesterol being the first molecule identified as having a direct role in the essential membrane merger (i.e. fusion) steps of fast, calcium-triggered exocytosis, as occurs under physiological conditions, in depolarized nerve terminals [3], [4]. In addition, as a microdomain (i.e. ‘raft’) organizer, cholesterol also plays a critical role in maintaining the localized presence of other components critical to targeting, priming, docking, and subsequent fusion [5]–[9].

Among the tools used to study cholesterol, perhaps the most widely applied has been methyl-ß-cyclodextrin (MβCD), a cyclic oligosaccharide that can deplete cellular membranes of cholesterol by increasing the water solubility of the sterol [10]. MβCD has thus been used with increasing frequency to examine the physiological roles of cholesterol. Studies utilizing MβCD have implicated cholesterol in mediating or modulating a wide range of membrane-associated cell properties and functions in a range of secretory cell types, including neurons. Such cellular processes include excitability [11]–[14], ion channel activity [15], [16], exocytosis [3], [4], [17]–[19], endocytosis / vesicle recycling [20], [21], neurotransmitter uptake and storage [22], [23] and postsynaptic receptor localization [16], [24]–[26]. Such studies make a case that cholesterol may be a critical membrane component for all the key steps in chemical synaptic transmission.

The sole use of MβCD to examine and identify cholesterol-dependent functions, however, poses at least two main limitations. First, it can be challenging to demonstrate definitively that a physiological effect is caused by a change in cholesterol level rather than some non-specific effect of MβCD, such as interaction with and removal of phospholipids [27], [28]. Some studies employing MβCD do not even report cholesterol levels, although many do provide corroborating evidence showing the same physiological effect with inhibitors of cholesterol synthesis, including an assessment of gross changes in the levels of cholesterol. Thus, the second limitation is that such qualitative assessments of global membrane cholesterol concentration may not correlate with the local concentrations at the functional sites of interest. The use of multiple pharmacological tools to confirm the direct role of cholesterol in any given mechanism thus remains the most secure approach. Why do these issues exist? As noted above, there is considerable evidence [29] that cyclodextrins exert pleiotropic effects on membranes, removing other lipidic (and protein) components, as well as depleting cholesterol from both fluid and cholesterol-enriched microdomains. Thus, effects of MβCD on physiological functions should be interpreted with caution and always confirmed with alternative approaches.

One such alternative approach to investigating cholesterol-dependent physiological effects is suggested by observations that thermal acclimatization for periods of weeks can alter the content of cholesterol and other lipids in ectothermic animals [30]. This raised the question of whether or not animals acclimatized to different temperatures, whose own metabolism would generate differences in levels of cholesterol and other membrane lipids, would respond similarly to cholesterol-depletion with MβCD. Thus, here we used acclimatization temperature as a tool to address both the acute effects of MβCD and the postulated roles of cholesterol in the physiology of chemical neurotransmission. We chose to address this question using crayfish, since these animals are known to adapt reasonably rapidly to decreases in temperature, assuming a more ‘quiescent’ state [31]. In addition, MβCD is recognized to elicit several presynaptic effects on crayfish neuromuscular preparations, including: (a) failure of impulses to propagate through axonal branches, (b) reduction in the amplitude of excitatory junctional potentials (EJPs), (c) modest enhancement of evoked transmitter release from directly stimulated synaptic terminals and (d) enhancement of spontaneous transmitter release [12]. Our results confirm that MβCD elicits presynaptic changes, but quantitatively show that MßCD also alters the responsiveness of postsynaptic cells. Both pre- and postsynaptic effects are modulated by thermal acclimatization. However, differential effects between thermally acclimatized groups indicate that some effects of MβCD correlate with its ability to decrease membrane cholesterol concentrations, but others do not. The complexity of the physiological effects, in particular during acute treatment with MβCD, suggest that more defined preparations and the thorough removal of MβCD are necessary to best assess (i.e. quantitatively) the potential roles of cholesterol itself in specific physiological functions.

## Results

### Effects of MβCD on synaptic transmission

**Figure 1:**
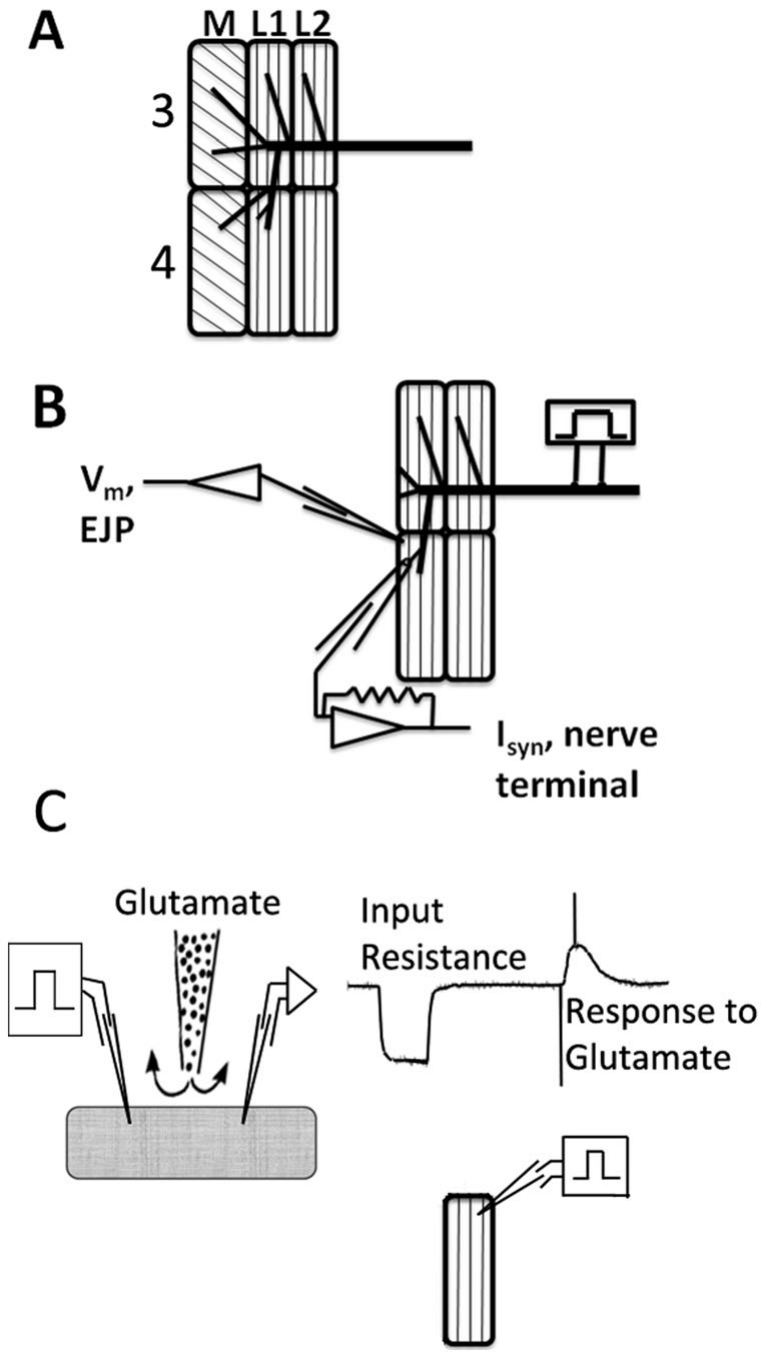
Experimental preparation. *A*. Deep abdominal extensor muscles M, L1 and L2 were exposed in abdominal segments 3 and 4. *B*. EJPs and synaptic currents were recorded using intracellular and loose patch electrodes, respectively. *C*. Iontophoresis was onto muscle segment 4 as close as possible to the neuromuscular junction.

**Figure 2 pone-0036395-g002:**
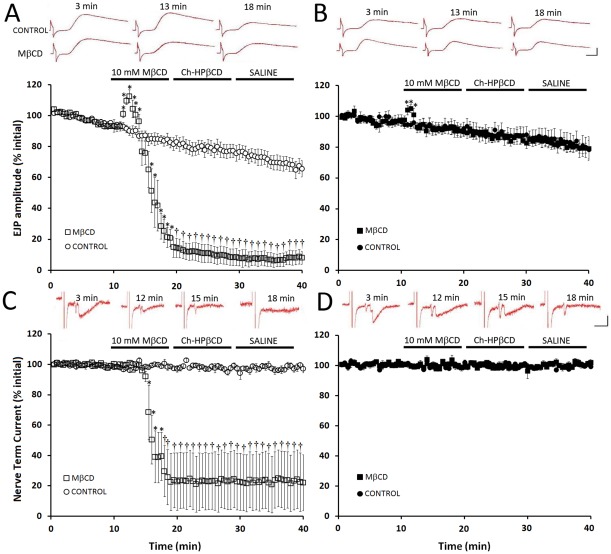
MβCD causes a reduction in EJPs which coincide with impulse failure only in neuromuscular preparations from cold-acclimatized animals. Intracellular recordings were made from L1 muscle fibers within the 4^th^ abdominal segment while simultaneously recording extracellularily through a loose patch electrode placed over a synapse. These recordings detect the propagating action potential (AP) invading the presynaptic terminal, the postsynaptic current following the AP, and an EJP. *A*. Cold-acclimatized group (open symbols). Control (no MβCD, open circles) depicts low-frequency depression, inherent during phasic stimulation. Application of 10 mM MβCD resulted in an initial significant (P<0.05) transient increase in EJP amplitude followed by a rapid reduction in EJP amplitude (P<0.05) which did not recover following the application of 10mM Ch-HPβCD or a subsequent saline wash. *B*. Warm-acclimatized group (closed symbols). Application of 10 mM MβCD resulted in an initial significant (P<0.05) transient increase in EJP amplitude, which quickly returned to control values, and remained stable for the remainder of the recording period. Inset (A–B) shows recorded signals of EJPs at selected time points. Scale bars: 5 mV, 5 ms. *C*. Extracellularily recorded nerve terminal currents from cold-acclimatized animals. The nerve terminal current remained stable during the pre-application period; application of 10 mM MβCD resulted in a loss of the nerve terminal signal in 6/7 trials, consequently producing a marked reduction in the amplitude of the nerve terminal current (P<0.05) which did not recover with the application of 10 mM Ch-HPβCD or a subsequent saline wash. *D*. Extracellularily recorded nerve terminal currents from warm-acclimatized animals. Application of 10 mM MβCD had no effect on the amplitude of the nerve terminal current. Inset (C–D) shows traces from loose patch recordings at selected time points. Initial downward deflection is the nerve terminal current, following by the post-synaptic current. Scale bars: 0.5 nA, 5ms. N = 7. * indicates P<0.05, † indicates P<0.01.

Effects of the cholesterol chelator, methyl-β-cyclodextrin (MβCD), on neuromuscular synapses were first assessed by recording excitatory junctional potentials (EJPs) in phasic abdominal extensor muscles (Fig. 1). At the stimulus frequency used (0.2 Hz) there was a gradual decrease in EJP amplitude over several minutes, due to low frequency depression [12], [32]; such depression was present in preparations from both cold- and warm-acclimatized crayfish and was modest (albeit more pronounced in the former group), decreasing EJP amplitude by ∼20–30% during the 40 min over which recordings were made Fig. 2A–B).

Application of 10 mM MβCD to preparations from cold-acclimatized crayfish (14°C) elicited two separate effects on EJP amplitude (Fig. 2A). The first was a small, transient increase approximately 3 min after beginning perfusion with MβCD, and the second was a dramatic decrease in EJP amplitude that occurred at 6–10 min of MβCD exposure. The early effect increased EJP amplitude by 18±4%, which was significantly different (P<0.05) from parallel control preparations not exposed to MβCD. At 10 min of MβCD exposure, EJP amplitude had decreased to ∼20% of the level observed before application (i.e. an 80% reduction), which was significantly different from the control group (P<0.05). The reduction in EJP amplitude could not be recovered either by washing for 10 min with cholesterol-saturated hydroxypropyl-β-cyclodextrin (Ch-HPβCD; in order to load exogenous cholesterol into the preparation) or by a subsequent saline wash (10 min). In contrast, application of 10 mM MβCD to preparations from warm-acclimatized (20–22°C) crayfish elicited an almost immediate transient increase in EJP amplitude of 11±2% followed by a return to baseline amplitude relative to parallel control preparations not exposed to MβCD (Fig. 2B). Thus, the effects of MβCD on EJP amplitude at room temperature depended on the temperature to which crayfish were previously acclimatized. Application of MβCD elicited a transient increase in EJP amplitude of ∼10–15%, although this was delayed in preparations from cold- vs. warm-acclimatized animals; in the continued presence of MβCD there was a subsequent reduction of EJP amplitude (13%/minute) only in preparations from the cold-acclimatized animals.

The reduction in EJP amplitude in preparations from cold-acclimatized crayfish is similar to that noted in an earlier report that found this effect correlated with failure of impulses to propagate to synaptic terminals [12]. These observations suggested that EJPs may become smaller because of failure of impulse propagation down axonal branch points, ultimately reducing the number of synaptic boutons releasing transmitter [18], [33], [34]. We recorded nerve terminal current with a loose patch electrode to determine whether or not the effects of MβCD on impulse propagation would be altered by acclimatization temperature in a manner consistent with the effects on EJP amplitude. Loose patch signals were acquired simultaneously with the intracellularly recorded EJPs to ensure that any lost signals were not due to failure of the stimulus to exceed threshold at the input site. The nerve terminal signals were typically biphasic, similar to many observed by Dudel [35] and were followed immediately by quantal events or failures. Propagation failure was indicated by loss of the nerve terminal signal and was always accompanied by complete loss of quantal events. Preparations from cold-acclimatized animals exhibited a dramatic decrease in the amplitude of nerve terminal current, which began after the initial 3 min of exposure to 10 mM MβCD (Fig 2C). The nerve terminal signal was completely lost in 6 of the 7 trials, and could not be recovered by washing with Ch-HPβCD nor with saline. In the remaining trial the nerve terminal current showed only a slight decrease in amplitude (5–16% decrease). The gradual reduction to 20% of initial current amplitude over the 10 min exposure to MβCD was caused by variations in the time at which nerve terminal signals were lost in different trials. Nerve terminal current was not lost in any preparations from warm-acclimatized crayfish during treatment with MβCD (Fig. 2D). Thus, the ability of MβCD to impair impulse propagation depended on the temperature to which crayfish were previously acclimatized and, at incubation times greater than ∼3 min, correlated with the reduction in EJP amplitude (Fig. 2A).

**Figure 3 pone-0036395-g003:**
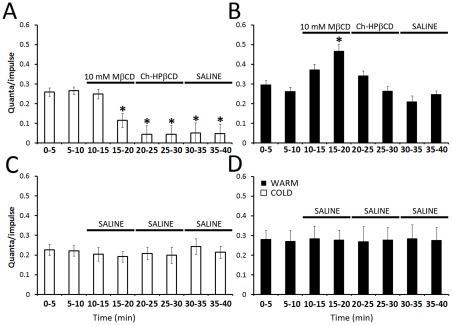
MβCD has opposing effects on quantal content in neuromuscular preparations from cold- vs. warm-acclimatized animals. Extracellular focal recordings were made from nerve branches within abdominal segment IV while stimulating the nerve in segment III. These recordings detected the presynaptic current and postsynaptic response following nerve stimulation. Quantal content was determined using the failures method. *A*. Cold-acclimatized group (open bars). First 10 min reflects saline perfusion. Application of 10 mM MβCD ultimately resulted in a significant reduction of quantal content after 10 min; this correlated with a reduction in EJP amplitude, and a loss of the nerve terminal signal. Quantal content could not be recovered with cholesterol loading (10 mM Ch-HPβCD) or a subsequent saline wash *B*. Warm-acclimatized group (closed bars). Application of 10 mM MβCD ultimately resulted in a significant increase in quantal content after 10 min. The increase in quantal content reversed following the application of 10 mM Ch-HPβCD, and stabilized to control levels during the saline wash. *C–D*. Control recordings from the cold- and warm-acclimatized groups, respectively, indicate stable quantal content over the 40 min recording period. (Stimulation: 0.2 Hz, data were averaged into 5 min bins, giving 60 stimuli per bin. N = 7. * indicates P<0.05.).

Failure of impulses to propagate to the nerve terminals and the concomitant reduction in EJP amplitude in cold-acclimatized crayfish correlated with a reduction in neurotransmitter release. Quantal content decreased by 54±10% (P<0.05) in preparations from cold-acclimatized crayfish at 10 min of exposure to 10 mM MβCD (Fig. 3A). Exposure to Ch-HPβCD did not recover quantal content, nor did a saline wash. In individual trials, the decrease in quantal content coincided with disappearance of the nerve terminal signal. No significant change in quantal content was observed at 3 min of exposure to MβCD, when EJP amplitudes were transiently elevated. In preparations from warm-acclimatized crayfish, quantal content increased by 50 ±4% at 10 min of exposure to MβCD (Fig. 3B; P<0.05). This finding is consistent with the fact that impulses were still able to reach the synaptic terminals (Fig. 2B), and with observations that the presence of MβCD increased transmitter release from synaptic boutons activated directly with electrical stimulation or with a calcium ionophore [12]. Subsequent exposure to Ch-HPβCD resulted in a return of quantal content to control levels, and a saline wash resulted in stable release comparable to controls. Control preparations not exposed to MβCD showed no significant change in quantal content, indicating that the level of transmitter release was stable in the neuromuscular preparations throughout the 40 min recording period, regardless of acclimatization temperature (Fig 3 C, D). Thus, over a 10 min incubation period, MβCD altered release of neurotransmitter from synaptic terminals, and the direction of the change depended upon acclimatization temperature. However, the increase in quantal content observed at 10 min of MβCD exposure in the warm-acclimatized group (Fig. 3B) was not accompanied by an increase in EJP amplitude (Fig. 2B). This suggested that MβCD might also affect factors downstream of transmitter release, such as input resistance of the muscle fibers or sensitivity of postsynaptic receptors to the neurotransmitter, L-glutamate [36], [37].

### Effects on muscle fibers

**Figure 4:**
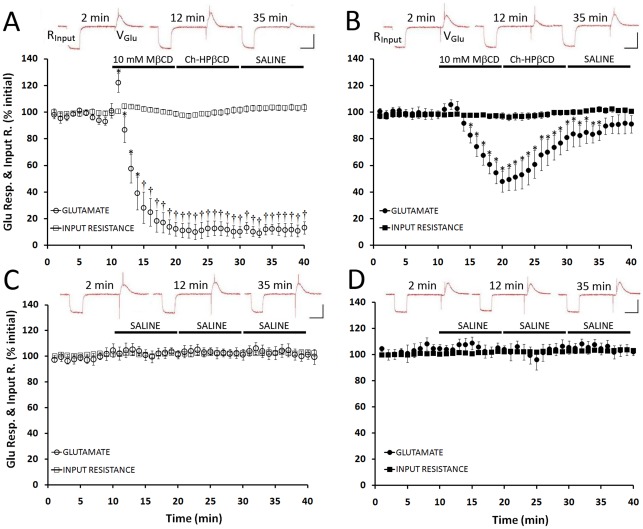
MβCD alters postsynaptic responsiveness. Intracellular voltage recordings were made from L1 muscle fibers within segment IV while iontophoretically applying L-glutamate and simultaneously injecting hyperpolarizing current intracellularly. These recordings detected changes in input resistance of muscle fibers as well as any changes in postsynaptic receptor sensitivity. Under no conditions was there an effect on the input resistance of muscle fibers (open and closed squares). *A*. Cold-acclimatized group (open symbols). Responsiveness to iontophoretically applied glutamate (open circles) revealed a significant (P<0.05) transient increase following the application of 10 mM MβCD, that subsequently resulted in a 17%/min reduction in the sensitivity to applied glutamate which plateaued within 6 min. Attempts to recover responsiveness, using a cholesterol-loaded cyclodextrin (Ch-HPβCD) and a saline wash, were unsuccessful. *B*. Warm-acclimatized group (closed symbols). While there was no immediate change in responsiveness to applied glutamate (closed circles), a subsequent gradual reduction during exposure to MβCD occurred. Application of Ch-HPβCD recovered responsiveness to 90% of initial values, and a subsequent saline wash resulted in a return to control values. *C-D*. Control recordings from cold- and warm-acclimatized groups were stable throughout the 40min recording period. Insets: recordings of input resistance and responsiveness to iontophoretically applied glutamate at selected time points. * indicates P<0.05, † indicates P<0.01. N = 7. Scale bars: 15 mV, 250 ms.

In preparations from cold-acclimatized crayfish, treatment with 10 mM MβCD elicited an almost immediate transient increase of 25±7% (P<0.05) in the amplitude of depolarization evoked by exogenous L-glutamate (Fig. 4A). This transient increase was followed immediately by a rapid drop (17% / min) in the amplitude of depolarization which plateaued within 6 min of exposure to MβCD; voltage responses decreased by 60±7% and by 90±3% at 5 min and 10 min of MβCD exposure, respectively, and the effect was statistically significant at both times compared to control preparations (P<0.01). The reduction in responsiveness to L-glutamate was not reversed by washing with 10 mM Ch-HPβCD (10 min) or subsequently with saline (10 min; Fig. 4A). In preparations from warm-acclimatized crayfish, MβCD appeared to elicit a small transient increase in responses to glutamate within 2–3 min of application (Fig 4B). When data at this time point were compared to responses over the entire pre-application period, the increase approached statistical significance (P = 0.056, ANOVA). Subsequently, the amplitude of glutamate responses decreased, but this did not begin until ∼2 min after exposure to MβCD and was both slower (7% / min) and less extensive (∼50 % inhibition after 10 min) than in the cold-acclimatized group (Fig 4B). In the warm-acclimatized group, the decreased responsiveness to L-glutamate was also largely reversed by washing in Ch-HPβCD (10 min), and reversed completely upon subsequent washing in saline (P<0.05).

MβCD had no effect on input resistance of the muscle fibers, estimated as cord resistance (Figs. 4A, B) or as slope resistance (Table 1), in either warm- or cold-acclimatized crayfish. Control preparations not exposed to MβCD showed no significant change in either input resistance or responses to L-glutamate over the recording time (Fig. 4C, D). MβCD did not alter assayed levels of glutamate in physiological saline (Figure S1) and, thus, did not appear to sequester glutamate.

**Table 1:** Input resistances in deep abdominal extensor muscle fibers, estimated using current pulses of varying amplitude (i.e. “slope” resistance).

	Control	MβCD
Cold-acclimatized	115+8 kΩ	107+10 kΩ
Warm-acclimatized	85+10 kΩ	78+11 kΩ

Input resistance was measured in the same fibers in 23% Ca^2+^ Saline (“Control”) and, subsequently, in the presence of 10 mM MβCD (N = 5 in all cases).

In the warm-acclimatized group the increase in quantal release in the presence of MβCD did not lead to any change in EJP amplitude. Since input resistance also did not change, it seems likely that the responsiveness of postsynaptic receptors was reduced. The effects of MβCD on responses to iontophoretic application of glutamate confirm that the responsiveness of the postsynaptic cell was reduced, but it is not clear if the change in sensitivity represents effects on junctional receptors or extrajunctional receptors. Unfortunately, spontaneous miniature synaptic currents are very rare in the deep abdominal extensor muscles, and we did not observe them. In our loose patch recordings however, we could generally distinguish single and multiple quantal events (as reported previously for the deep abdominal extensor muscles [38]). To address this, we measured the amplitudes of evoked quantal currents corresponding to single events (Figure S2). In the warm-acclimatized group, quantal amplitude decreased by ∼10% (P<0.05) after 5–10 min exposure to MβCD and the effect reversed with cholesterol loading. In the cold-acclimatized group quantal size also decreased by ∼10% (P<0.05) but the effect did not reverse with cholesterol loading as impulse propagation did not recover.

### Lipid analyses

**Figure 5:**
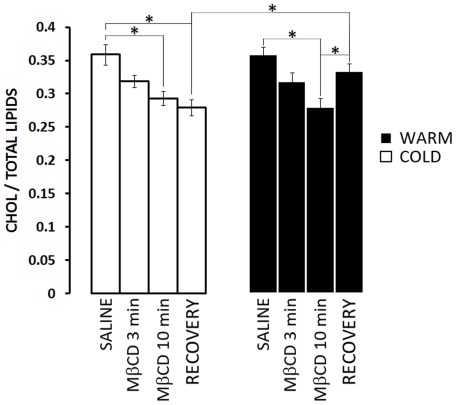
MβCD caused comparable significant cholesterol depletion in warm- and cold-acclimatized groups but this effect was irreversible in the latter. Cholesterol concentrations were assessed in the same neuromuscular preparations used for the electrophysiological analyses (Figures 1–4). Cold-acclimatized groups (open bars). Application of 10 mM MβCD resulted in a significant reduction in cholesterol after 10 min. Attempts to recover cholesterol by perfusion with Ch-HPβCD (RECOVERY) were unsuccessful. Warm-acclimatized groups (solid bars). Application of 10 mM MβCD resulted in a significant reduction in cholesterol after 10 min, comparable to that measured in the cold-acclimatized group. Subsequent perfusion with Ch-HPβCD resulted in recovery to a level comparable to the saline control group (i.e. baseline). N = 7. * indicates P<0.05.

The removal of cholesterol by MβCD, and the selectivity of this effect were examined by extracting and assaying total lipids from the neuromuscular preparations. MβCD reduced total cholesterol levels in neuromuscular preparations from both cold- and warm-acclimatized crayfish without altering the level of any other lipids analysed (phosphatidic acid, phosphatidylcholine, phosphatidylserine, phosphatidylethanolamine and phosphatidylinositol; Figure S3) except for sphingomyelin in the cold-acclimatized animals (9±1% reduction). Preparations from both cold– and warm-acclimatized crayfish showed a trend toward reduced cholesterol concentrations within 3 min of MβCD exposure, and by 10 min of treatment there was a significant decrease in the total cholesterol in both cold and warm-acclimatized groups (Fig. 5; P<0.05). Both acclimatization groups had equivalent proportions of cholesterol at the beginning of the experiment (when exposed only to saline), suggesting that total cholesterol levels in full neuromuscular preparations at these acclimatization temperatures were not significantly altered. Notably, phosphatidylethanolamine levels were significantly higher in the cold- vs. warm-acclimatized group (Figure S3). Proportions of cholesterol were also nearly identical between the two acclimatization groups at the end of the 10 min exposure to MβCD, indicating that the extent of cholesterol chelation was comparable in both cases. The two groups, however, differed following attempts to restore cholesterol to the cells. Washing for 10 min in Ch-HPβCD failed to increase cholesterol in the cold-acclimatized group but did increase cholesterol significantly in the warm-acclimatized group (P<0.05). Following the saline wash, cholesterol was significantly higher in the warm-acclimatized group than in the cold-acclimatized group (P<0.05).

**Figure 6:**
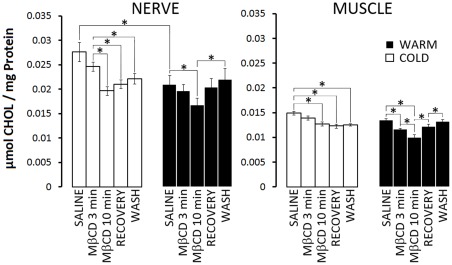
Cholesterol content, depletion, and recovery differ significantly between cold- and warm-acclimatized groups: comparison of isolated nerve and muscle. Cholesterol concentrations were assessed independently in both isolated nerve and muscle tissue. Lipids were extracted separately from isolated nervous tissue (ventral nerve cords from the crayfish abdomen) and isolated muscles. Nerve (left): initial concentrations of cholesterol were significantly reduced in warm- vs. cold-acclimatized animals. Cold-acclimatized group (open bars). Application of 10 mM MβCD caused a significant reduction in cholesterol after 10 min. Attempts to recover cholesterol by perfusion with Ch-HPβCD (recovery) and a saline wash were unsuccessful. Warm-acclimatized group (solid bars). Application of 10 mM MßCD also caused a significant reduction in cholesterol after 10 min. Subsequent perfusion with Ch-HPβCD and saline resulted in recovery of cholesterol to initial levels (i.e. baseline). Muscle (right): initial cholesterol levels were comparable in both the cold- and warm-acclimatized groups. Application of 10 mM MβCD caused a significant reduction in cholesterol after 10 min regardless of the acclimatization condition. Again, attempts to recover cholesterol using Ch-HPβCD and a saline wash were unsuccessful in the cold-acclimatized group. Notably, in the warm-acclimatized group, 3 min exposure to MβCD already resulted in a significant reduction of muscle cholesterol. Perfusion with Ch-HPβCD and saline was sufficient to return cholesterol levels to initial (i.e. baseline) levels in the warm-acclimatized group. N = 7.* indicates P<0.05

Due to differences in cellular mass, the neuromuscular preparations were comprised mainly of muscle fibers and only small amounts of nervous tissue. To determine whether MβCD might have different effects on nerve and muscle, lipids were extracted separately from isolated nervous tissues (ventral nerve cords from the crayfish abdomen) and isolated muscles from which the nerves and their branches had been cut away (Fig. 6). It is immediately striking that there is substantially more cholesterol in nerve than in muscle, regardless of acclimatization temperature, and that there is 24±3% more cholesterol in nerves from cold- relative to warm-acclimatized crayfish. The results showed essentially the same trends as the neuromuscular preparations (Fig. 5). Exposure to MβCD for 3 min showed a trend toward cholesterol reduction that was statistically significant in muscles from warm-acclimatized crayfish, and a 10 min exposure to MβCD reduced cholesterol significantly in all tissues. Subsequent washing with Ch-HPβCD and saline brought cholesterol back to control levels in tissues from warm-acclimatized crayfish but failed to cause statistically significant recovery in tissues from cold-acclimatized crayfish.

## Discussion

In the present work acclimatization temperature was used as a tool to assist in understanding the effects of MβCD on chemical synapses and to provide insight into how extensively these effects are related to the depletion of cholesterol from cell membranes. Multiple pre- and post-synaptic effects, including acute effects of MβCD that are not linked to the extraction of cholesterol, complicate efforts to understand the synaptic release mechanism. Direct assay of cholesterol in the neuromuscular preparations used for electrophysiological assessments, as well as in separate neural and muscular tissue, has dissociated some effects of MβCD from the depletion of cholesterol. It is thus critical to control for the acute effects of MβCD as a reagent in dissecting the physiological roles of cholesterol in different molecular mechanisms.

MβCD

While most cyclodextrins exhibit some ability to remove different lipids from membranes, MβCD has reasonable selectivity for cholesterol, although this can vary somewhat depending on the actual composition of the membrane. Importantly, it is now apparent that interfacial effects including adsorption, local membrane destabilization, and desorption of the cholesterol-cyclodextrin complex from the membrane interface (i.e. removal of cholesterol) occur on very rapid timescales (i.e. hundreds of nanoseconds) and overcome substantial energy barriers [39]. Thus, after this critical adsorption of MβCD to the membrane and its subsequent desorption with cholesterol, the sterol is complexed (i.e. ‘solubilized’), establishing a dynamic equilibrium between membrane and ‘soluble’ cholesterol during which time cholesterol can also be inserted at other membrane locations. The removal of cholesterol will yield a net decrease in local negative curvature and also result in the rapid equilibration of remaining cholesterol from the inner to the outer monolayer; thus, there are more structural effects on the membrane than simply the physical removal of cholesterol. It is therefore important to realize that there are acute effects of MβCD–membrane interactions as well as dynamic responses to cholesterol removal, and both must be considered in addition to the effect of a net reduction in membrane cholesterol. Interpretation must thus also take into account the possibility of cholesterol re-insertion into the membrane from the ‘soluble’ pool.

### Acclimatization temperature alters the effects of MβCD

We have established quantitatively that treatments with MβCD result in the selective extraction of cholesterol from the membranes of crayfish neuromuscular preparations, irrespective of previous acclimatization temperature. This confirms previous observations using filipin imaging on preparations from crayfish acclimatized at 15–18°C [12]. There were however several differences between the effects of MβCD on preparations from warm- and cold-acclimatized animals when these were assessed at the same temperature (21°C). First, although application of MβCD elicited a small, rapid, transient increase in EJP amplitude in both groups, this effect was almost immediate in the warm-acclimatized group but delayed in the cold-acclimatized group (Fig. 2). Second, continued (6–10 min) exposure to MβCD elicited a dramatic decrease in EJP amplitude and failure of impulse propagation to synaptic terminals in the cold-acclimatized group, but neither of these effects occurred in the warm-acclimatized group (Fig. 2). Third, extended treatment (i.e. 10 min) with MβCD decreased quantal release in the cold-acclimatized group but increased release in the warm-acclimatized group (Fig. 3), despite a comparable decrease in the amplitude of evoked single quantal events in both groups (Figure S2); both effects in the cold-acclimatized group were irreversible. Fourth, application of MβCD yielded a rapid, transient increase in the amplitude of muscle depolarization evoked by exogenous L-glutamate, but this effect was only significant in the cold-acclimatized group (Fig. 4). Finally, although exposure to MβCD subsequently decreased responses of muscle fibres to L-glutamate in both experimental groups, the effect was more pronounced and irreversible in preparations from cold-acclimatized animals. Results from direct, quantitative lipid analyses enabled assessment of whether some or all of these differences related to cholesterol levels. The ability of MβCD to reduce transmitter release in the cold-acclimatized but not in the warm-acclimatized group was most likely related to its effect(s) on impulse propagation. Impulses failed to propagate to the synaptic terminals in preparations from cold-acclimatized crayfish after 6-10 min of exposure to MβCD, when cholesterol levels were significantly reduced. In the warm-acclimatized group, however, impulse propagation was not disrupted, even though cholesterol levels were significantly reduced by the end of the exposure to MβCD.

### Cholesterol levels

Despite little difference in the ratio of cholesterol to total lipids in neuromuscular preparations from cold and warm-acclimatized crayfish, analyzing cholesterol levels separately in nerve and muscle revealed substantially more cholesterol in nerves, and comparison between the warm- and cold-acclimatized groups revealed significantly less neuronal cholesterol in the warm-acclimatized group (Fig. 6). As cholesterol is important in synapse formation [40] and is a major component of secretory vesicles [41], [42], the difference in cholesterol content between nerve and muscle tissues is perhaps not surprising. Similarly, it is known that cold-acclimatization can result in increased levels of membrane cholesterol in some ectothermic animals [8], [30].

Notably, in preparations from cold-acclimatized crayfish, treatment with MβCD caused little change (15±3 %) in cholesterol levels from muscle, but a substantial change (29±4%) in cholesterol levels from nervous tissue. In preparations from warm-acclimatized crayfish, this effect reversed; only in muscle from warm-acclimatized animals did 3 min of exposure to MβCD significantly decrease cholesterol levels. Furthermore, in both muscle and nerve, previous cold-acclimatization resulted in an inability to take-up exogenous cholesterol; warm-acclimatized preparations depleted of cholesterol were readily restored to native cholesterol levels using a standardized protocol for the delivery of exogenous cholesterol [3], [4].

### Transient increases in EJP

The transient increase in EJP amplitude (18%) in the cold-acclimatized group occurred within 1-3 min of exposure to MβCD, preceding both impulse propagation failure and any significant reduction in cholesterol levels in nerve or muscle tissue. The comparable transient increase in EJP amplitude in the warm-acclimatized group (11%) occurred within seconds of MβCD application, substantially preceding any other effects, including cholesterol depletion. Thus, if these transient increases in EJP amplitude are not directly linked to the loss of cholesterol, there must be another effect of acute exposure to MβCD. Considering the rapid interfacial effects of cyclodextrin adsorption [39], this would indicate that the addition of MβCD yielded localised destabilisation resulting in the fusion of some vesicles (or perhaps transient leakage of transmitter). Whether this rapid, transient increase in EJP is directly attributable to cholesterol removal and/or to localized interfacial effects remains to be assessed by methods with more spatial and temporal resolution, likely coupled with molecular dynamic analyses [39].

Since there was no significant increase in quantal content or muscle fiber input resistance within 1–3 min of exposure to MβCD, it also seemed likely that the transient increase in EJP amplitude was related to some extent to an increase in the sensitivity of postsynaptic receptors to the transmitter. Notably, a similar rapid, transient increase in the amplitude of glutamate-evoked depolarization in muscle was also seen in response to the application of MβCD. This transient increase was statistically significant in cold-acclimatized preparations (25%, P<0.05) and approached statistical significance in warm-acclimatized preparations (8%, P = 0.056; Fig. 4). Transient increases in responses to glutamate can be caused by a decrease in desensitization of glutamate receptors, as reported for Concanavalin A in locust skeletal muscle [43]. Thus, it is possible that MβCD might reduce desensitization to the transmitter, but we cannot rule out other possible explanations, such as receptor insertion or redistribution [44]–[46]. Since the apparent increase in glutamate sensitivity was just below statistical significance in the warm-acclimatized group, we cannot rule out the possibility that MβCD might enhance transmitter release transiently in these preparations (as discussed above). Such an effect, however, would be small, since EJP amplitude increased by only 11%. We thus interpret the transient effects of MβCD on EJP amplitude to represent transient disruptions of the plasma membrane due to initial interactions with the cyclodextrin, which may or may not result in local changes in cholesterol level. Such potentially non-specific, interfacial effects of MβCD should thus be taken into account whenever physiological assessments are carried out in the presence of this cyclodextrin. There is clearly a difference between assessing the acute effects of MβCD as a reagent and the role of cholesterol in molecular mechanisms underlying specific physiological changes [3], [4], [9].

### Cholesterol, EJPs and quantal content

In contrast to the rapid transient increases in EJP amplitude seen early after the addition of MβCD, longer treatments (i.e. 6–10 min) resulted in significant depletion of nerve and muscle cholesterol from both acclimatization groups. However, this also resulted in opposing effects on the quantal content from the two acclimatization groups (Fig. 3). Thus, the use of two different acclimatization conditions effectively dissociated quantal content from total membrane cholesterol levels.

The removal of cholesterol would result in a net increase in local positive curvature that would not be conducive for membrane fusion [3], [4]; this likely contributed in part to the reduction of EJPs seen in the cold-acclimatized group as the exposure to MβCD continued (Fig 2A). However, as membranes in the warm-acclimatized group readily integrated exogenous cholesterol, it seems likely that they would also efficiently re-capture some of their own cholesterol from MβCD complexes which would contribute to the increased quantal content observed in the warm-acclimatized group (Fig. 3B).

However, the biggest contributor to the reduction in EJP amplitude in the cold-acclimatized group is most likely related to the observed decline in impulse propagation to synaptic terminals, preventing transmitter release (Figs 2 and 3). We suspect that the reductions in EJP amplitude mainly reflect the progressive loss of active axonal branches and, thus, progressive loss of secretion from synapses on each muscle fibre. Failure of impulse propagation in crayfish nerve terminals with repeated stimulation has been shown to occur at distal branch points and to spread toward the primary axon [47]. This interpretation is consistent with the reported loss of nerve terminal signal prior to loss of impulse in the primary axon during treatment with MβCD [12]. Since decreased levels of cholesterol have been linked to decreased functional efficacy of ion channels [15], [48], [49], it is tempting to suggest that impulse propagation failure correlates with changes in cholesterol. Indeed, our cholesterol measurements, and previous work with filipin staining [12], indicate that changes in impulse propagation correlate with reduced cholesterol levels, but only in cold-acclimatized animals. Impulse propagation is not impaired in the warm-acclimatized group even though MβCD depletes these preparations of cholesterol. Thus, our results suggest that the ability of MβCD to impair impulse propagation depends on factors associated with prior acclimatization to a lower temperature.

In the warm-acclimatized group, where impulse propagation was not impaired, MβCD enhanced transmitter release after 5–10 min. This effect was consistent with previous observations that MβCD can enhance release if synaptic terminals are excited electrically [12]. Enhancement of release in our trials reversed during subsequent treatment with Ch-HPβCD, suggesting that this effect may involve cholesterol removal. This interpretation, however, remains inconsistent with the bulk of literature indicating that cholesterol loss inhibits the release process (3, 4, 8 and 9+ others). Moreover, we have not ruled out the possibility that removal of MβCD itself may have reversed the enhancement of release in our trials.

The apparent increase in transmitter release / quantal content in the warm-acclimatized group, without a concomitant increase in EJP amplitude, could be due to reduced sensitivity of postsynaptic receptors to the transmitter [50]. This is consistent with the partial decline in post-synaptic responsiveness to glutamate in the warm-acclimatized group (Figure 4B) and a significant reduction in the amplitude of single, evoked quantal currents (Figure S2). Muscle fiber input resistance did not change and, thus, did not contribute to changes in EJP amplitude. Considering the pleiotropic effects of MβCD, additional effects on the pre- and post-synaptic membranes cannot be ruled out. However, a critical role for cholesterol in the post-synaptic response is clear: after the washout of MβCD, reduced cholesterol levels correlate with reduced responsiveness in the cold-acclimatized group. Furthermore, washout of MβCD and supplementation with exogenous cholesterol correlate with the full recovery of responsiveness in the warm-acclimatized group only. Thus, the mechanism underlying changes in postsynaptic sensitivity to glutamate is likely to involve changes in cholesterol and not loss of a critical protein.

### Postsynaptic effects

In contrast with an earlier report indicating that MβCD did not alter the size or shape of miniature end-plate potentials (mepps) in crayfish dactyl opener muscle [12], our data indicate that MβCD affects postsynaptic cells. Although it did not alter input resistance in muscle fibers, MβCD dramatically reduced responses to local application of L-glutamate by iontophoresis, suggesting a direct effect on glutamate receptor function. In the cold-acclimatized group, the decreased sensitivity of postsynaptic receptors to glutamate is likely to contribute substantially to the decrease in EJP amplitude. Taken as a whole, the ability of MβCD to alter so many synaptic properties makes it difficult to understand the acute effects of the compound, or to predict how it ultimately alters the overall input/output relation at chemical synapses.

As noted above, exposure to MβCD decreased responses to L-glutamate in both experimental groups at times that coincided with reduction in cholesterol levels in muscle cells. In the warm-acclimatized group, glutamate-sensitivity returned toward normal levels with the addition of exogenous cholesterol (Fig 3B). In the cold-acclimatized group, Ch-HPβCD failed to restore cholesterol to the cells, and responsiveness to glutamate failed to return. Thus, the ability of MβCD to impair glutamate responsiveness correlated well with its ability to remove cholesterol, suggesting that this postsynaptic effect is mediated at least in part by cellular depletion of cholesterol. The reduction in glutamate responsiveness, however, was greater in the cold-acclimatized group, even though the warm-acclimatized group exhibited a slightly greater degree of cholesterol depletion and thus slightly lower cholesterol levels. Thus, factors other than just cholesterol depletion likely influence the effect of MβCD on glutamate responsiveness.

The inability to recover EJPs, nerve terminal signals or cholesterol in the cold-acclimatized group is intriguing, particularly because these physiological effects recovered with cholesterol loading in other crayfish neuromuscular junctions [12]. In that report, as in ours, crayfish were maintained below room temperature, but experiments on isolated preparations were carried out at room temperature. The acclimatization temperatures, however, were slightly different (13–14°C in the present report and 15–18°C in [12]). At face value, these observations suggest that reducing acclimatization temperature makes it more difficult to restore cholesterol to plasma membranes following its removal with MβCD. It is also curious that acute exposure to MβCD impairs impulse propagation at room temperature in cold-acclimatized animals but not in animals acclimatized to room temperature, despite its ability to remove cholesterol in both cases.

The differential effects of MβCD on physiological responses from the cold- and warm-acclimatized groups may provide some insight into the functional nature of cholesterol packing or domains. For example, why is the transient rise in EJP amplitude delayed in the cold-acclimatized group following the application of MβCD (Fig. 2) yet the transient increased sensitivity to glutamate is almost instantaneous (Fig. 4)? First, the cold-acclimatized group have substantially more cholesterol in their nerves than do the warm-acclimatized group. Thus, at the same dose, MβCD would have to remove far more bulk cholesterol from the cold-acclimatized group before beginning to effectively impinge upon the functionally critical pool of cholesterol. In contrast, in muscle, there is little difference in the cholesterol content between cold- and warm-acclimatized animals. Furthermore, exogenous cholesterol can be efficiently incorporated into membranes from warm- but not cold-acclimatized animals (Figures 5, 6). Together, we interpret these effects to indicate substantial differences in lipid packing. In membranes from the cold-acclimatized animals, most cholesterol may be in larger microdomains that stably localize functionally critical proteins; thus, upon removal of cholesterol, domains rapidly dissipate resulting in pronounced effects on function. It is generally accepted that the major lipidic constituents of these microdomains are cholesterol and sphingomyelin. It is noteworthy that the only other significant change in lipid composition following MβCD application was an ∼10% reduction in sphingomyelin in cold-acclimatized preparations (Figure S3). This may at least in part account for the inability of these tissues from cold-acclimatized animals to take-up exogenous cholesterol and recover functional microdomains. We note, however, that this is further evidence of the pleiotropic effects of MβCD, and urge multiple controls and quantitative assessments of cholesterol in experiments involving this compound.

Acclimatization temperature has thus proven to be another important tool to understand the roles of cholesterol in specific physiological processes, and to highlight the non-specific or acute effects of treating cellular membranes with MβCD. In order to use MβCD most effectively to study the roles of cholesterol it is thus important that it be removed from the assay system prior to assessing functional and molecular changes; only this will ensure that correlations with membrane cholesterol content alone are being analyzed, and that any other potential molecular alterations can be identified under equilibrium conditions. The distinction is between assessing the effects of MβCD as a surface-active reagent vs. rigorously understanding the roles of cholesterol in physiological mechanisms.

## Materials and Methods

### Crayfish maintenance and dissection

*Procambarus clarkii* were obtained from Atchafalaya Biological Supply (Raceland, Louisiana, USA) and had carapace lengths ranging from 1 ½ to 3 cm. They were placed randomly into two holding tanks, one maintained at room temperature (20–22°C) and the other maintained at 14°C. Crayfish were allowed to acclimatize to these temperatures for 17–35 days, and were maintained in a room with 12∶12 light/dark cycle during the entire time course of the study; they were fed artificial crabmeat (Selection™ crab flavoured Pollack, from a local grocery store) three times per week.

Crayfish were cold anaesthetized immediately before being euthanized. The dorsal side of the abdominal shell containing the extensor muscles was removed and cut down the midline, and each half (Fig. 1A) was placed immediately in crayfish saline containing 50% of the normal calcium concentration and ten times the normal magnesium concentration (“50% Ca^2+^ Saline”, Table 2) based on physiological concentrations [51]. Each half of the abdomen was pinned, ventral side up, in a Petri dish lined with Sylgard™ for further dissection. Abdominal segments I, II, VI, and half of V were removed, and all of the medial (M) muscles in the remaining segments were removed. The remaining segments were pinned ventral side up in a rectangular recording chamber with dimensions 19 mm×12 mm×3 mm, which was filled with 50% Ca^2+^ saline to a volume of 684 microlitres. The preparation was pinned so as to stretch the lateral (L1 and L2) muscles and expose the nerve in segment III. A peristaltic pump was utilized to enable the efficient exchange of experimental solutions from the recording chamber at a rate of 800microliters/min.

**Table 2:** Chemical composition of salines used. All values are expressed in mM.

	50% Ca^2+^ Saline	26% Ca^2+^ Saline	Ca^2+^- free Saline	23% Ca^2+^ Saline	MβCD
NaCl	200.7	200.7	200.7	200.7	200.7
KCl	5.4	5.4	5.4	5.4	5.4
CaCl_2_.2H_2_O	6.5	3.4	-	3.1	3.1
MgCl_2_.6H_2_O	12.3	15.7	19.0	16.0	16.0
HEPES	5.0	5.0	5.0	5.0	5.0
MβCD	-	-	-	-	10.0

### Electrophysiological recording

All electrophysiological recordings as well as iontophoresis were carried out at room temperature (21°C). Excitatory junctional potentials (EJPs) and synaptic currents were recorded as described by Mercier and Atwood (1989). A suction electrode was utilized to stimulate the nerve in segment III, and recordings were made from muscles L1 in segment IV (Fig. 1B) to ensure that EJPs were elicited by activating excitatory axon #3 [52]. Stimuli were generated by a Grass (West Warwick, U.S.A.) S88 stimulator, fed through a Grass SIU5 stimulus isolation unit, and were delivered at a frequency of 0.2 Hz. EJPs were recorded using glass microelectrodes filled with 3 M KCl, connected to an intracellular electrometer (Warner Instruments, St. Laurent, Quebec, Canada). Tip resistance was typically 15 MΩ. Resting membrane potential (RMP) was recorded at the beginning and end of each trial.

Synaptic currents and nerve terminal signals were recorded using loose patch electrodes [53] with an outer diameter of 60 micrometers and an inner diameter of 10–15 micrometers, which were connected to a virtual ground circuit that lacked the capacity for direct stimulation through the loose patch electrode [54]. Loose patch signals were recorded simultaneously with EJPs. Quantal content was estimated using the failures method [55], according to the equation m  =  ln(N/N_o_), where m is the quantal content, N is the number of stimuli in the trial and N_o_ is the number of failures. Data were averaged into 5 min bins giving 60 stimuli per bin. Concentrations of calcium and magnesium in the extracellular saline were adjusted (Table 1) to ensure that failure rate was sufficiently high in each trial for the number of evoked quantal currents to fit a Poisson distribution [55], [56]. At least 10 min was allowed for the solution to wash though the recording chamber before beginning each experiment. Trials with Methyl-Beta-cyclodextrin (MßCD) were performed in the 23% Ca^2+^ crayfish saline.

Electrophysiological signals were monitored on a storage oscilloscope. Signals from the intracellular and loose patch amplifiers were digitized and acquired into data files using a computerized data acquisition system equipped with data analysis and recording software (Electronics Division, Brock University, St. Catharines, Ontario, Canada)[57]. Nerve terminal signals and EJPs were signal-averaged over 30s intervals, with each signal representing the average of 6 successive responses. The number of stimuli failing to elicit quantal currents was assessed by viewing stored recordings, which were also processed automatically to detect peak values for each signal (nerve terminal, quantal, and EJP). Peak values were stored in a.txt file and imported into Microsoft Office Excel 2007 for graphical and statistical analyses.

Input resistance was measured by inserting two glass microelectrodes filled with 3 M KCl into the same muscle fiber, injecting hyperpolarizing current through one microelectrode and recording voltage responses with the second. The electrodes were connected to separate electrometers (Warner Instruments, St. Laurent, Quebec, Canada), at least one of which was equipped with a bridge circuit for passing current. Injected currents were 150 ms in duration and were applied at a rate of 0.1 Hz. In the same trials, L-glutamate was applied to the muscle fibers iontophoretically through an extracellular microelectrode filled with 1 M L-glutamate. Iontophoretic current was applied through a Cyot721 Electrometer (World Precision Instruments, Inc., Sarasota, Florida, U.S.A.) using a pulse duration of 50 ms. To estimate cord resistance, current was injected into the muscle fibers 450 ms before each iontophoretic application of L-glutamate. In a separate set of trials, slope resistance was estimated by injecting a series of hyperpolarizing currents of varying amplitude, recording voltage responses and estimating the slope of voltage vs. current plots. Signals were acquired using the computerized data acquisition system, and signals were processed automatically to identify the amplitude of the glutamate response as well as the amplitude of the response to hyperpolarizing current.

MßCD and hydroxypropyl-beta-cyclodextrin (HPßCD)(Sigma-Aldrich Inc., Oakville, Ontario, Canada) were stored in powder form at room temperature and were dissolved in crayfish saline to a final concentration of 10 mM in each case. Cholesterol loaded HPßCD (Ch-HPßCD) was prepared as described previously [3]. The solutions were stirred for 30 min prior to and throughout each trial. All solutions were adjusted to pH 7.4.

### Lipid analyses

Tissues were snap frozen in liquid nitrogen at various stages of experimental treatment (i.e. before, during, and after exposure to MßCD) and stored at −80°C. Samples were manually homogenized on ice with a polyethylene pestle in a 1.5 mL tube with saline buffer (51.3 mM NaCl, 5 mM 4-(2-hydroxyethyl)-1-piperazineethanesulfonic acid (HEPES), 4.0 mM CaCl_2_, 1.7 mM KCl, 1.5 mM MgCl_2_; pH 7.9). The samples subsequently underwent lipid extraction according to the method of Bligh and Dyer [58], with previously described modifications [4], and were split into two aliquots. One aliquot was dissolved in 1×Reaction buffer (for enzymatic cholesterol determination: 50 mM NaCl, 5 mM cholic acid, 0.1% Triton X-100, 100 mM potassium phosphate pH 7.4), and the second aliquot was stored under N_2_ (−30°C) until required for phospho- and neutral lipid analyses. For automated high performance thin layer chromatography (HPTLC), lipids were dissolved into chloroform: methanol (2∶1,v/v) and loaded onto silica gel 60 HPTLC plates (EMD Chemicals, Darmstadt, Germany) with a parallel dilution series of lipid standards. All lipid standards were the oleic acid (18∶1Δ9) esters (Avanti Polar Lipids, Alabaster, Alabama, U.S.A.). Cholesterol in the samples was determined using the Amplex Red cholesterol assay kit (Invitrogen, Carlsbad, California, U.S.A.) according to the manufacturer's instructions. Protein content was assessed using a BCA protein assay (Thermo Scientific, New Hampshire, U.S.A.).

HPLC plates were prewashed with methanol: ethyl acetate (6∶4, v/v) and activated at 110°C for at least 30 min prior to loading. Dilution series of both native extract and lipid standards in chloroform: methanol (2∶1) were loaded onto HPTLC plates under N_2_ using the CAMAG Linomat IV, and developed in the CAMAG AMD2 (CAMAG Inc., Muttenz, Switzerland) as described elsewhere [3], [4], [7], [42]. For neutral lipids, HPTLC plates were developed in five steps: with dichloromethane: ethyl acetate: acetone (80∶16:4 v/v/v) to 40 and 55 mm, then sequentially with hexanes: ethyl acetate to 68 mm (9010 v/v), 80 mm (955 v/v), and 90 mm (100∶0 v/v). Phospholipids were resolved using a two-step-separation: to 90 mm with dichloromethane: ethyl acetate: acetone (80∶16∶4 v/v/v), dried under vacuum for 6 min, then developed again to 90 mm with chloroform: ethyl acetate: acetone: isopropanol: ethanol: methanol: water: acetic acid (30∶6∶6∶6∶16∶28∶6∶2, v/v).

Plates were sprayed uniformly with 10% copper sulfate (Copper (II) sulfate heptahydrate, Sigma Chemical Co., St. Louis, Missouri, U.S.A.) in 8% aqueous phosphoric acid, allowed to dry 10 minutes at room temperature, and then charred at 140°C for 10 minutes. Densitometric detection of charred plates was carried out using the CAMAG TLC Scanner 3 (CAMAG Inc, Wilmington, North Carolina, U.S.A.). Fluorescence was detected using the ProExpress multiwavelength imager (Perkin Elmer, Boston, Massachusetts, U.S.A). Copper sulfate signals were assessed with excitation and emission of 540/30 and 590/20, respectively [7], [42]. Digital images of the chromatograms were analyzed using ImageQuant 5.2 (GE Healthcare, Piscataway, New Jersey, U.S.A.)

### Glutamate Assay

Glutamate was assayed using a glutamate dehydrogenase catalyzed reaction in which glutamate is oxidized and NADH is formed and coupled to a formazan (MTT)/phenazine methosulfate reagent [59]. An assay kit (BioAssay Systems, Hayward, CA) was used according to the manufacturer's instructions.

### Statistics

For EJP and iontophoresis, statistical significance was determined using a Student's t-test at each time point. Statistical significance for lipid analyses were determined using a one-way ANOVA. Statistical significance for differences in quantal content was determined using a Mann-Whitney U test.

## Supporting Information

Figure S1 **MβCD does not sequester glutamate.** Glutamate concentrations were assayed at various concentrations to determine if the reduction in responsiveness to iontophoretically applied glutamate was due to chelation/sequestration by MβCD. Glutamate concentrations were not altered by the presence of MβCD.
(TIF)

Figure S2 **MβCD significantly decreased the amplitude of evoked single quantal events in both acclimatization groups.** Extracellular focal recordings were made from nerve branches within abdominal segment IV while stimulating the nerve in segment III. These recordings detected the presynaptic current and postsynaptic response following nerve stimulation. We used the amplitude of single quantal events to determine postsynaptic changes. *A*. Cold-acclimatized group (open bars). First 10 min reflects saline perfusion. Application of 10 mM MβCD resulted in a significant reduction in the amplitude of single quanta. Since the nerve terminal signal was lost in 6 of 7 trials of the cold-acclimatized group, not enough data could be generated to accurately determine the amplitude of single quantal events after 5 min of MβCD. *B*. Warm-acclimatized group (closed bars). Application of 10 mM MβCD ultimately resulted in a significant decrease in amplitude of single quanta after 10 min. *C-D*. Control recordings from the cold- and warm-acclimatized groups, respectively, indicate stable single quantal size over the 40 min recording period. (Stimulation: 0.2 Hz, data were averaged into 5 min bins, giving 60 stimuli per bin. N = 7. * indicates P<0.05.)
(TIF)

Figure S3 **MβCD does not significantly alter the concentrations of other phospholipids in the neuromuscular preparation.** Relative concentrations of phospholipids assessed from the same neuromuscular preparation used for the electrophysiological analyses. MβCD did not alter the level of any of the major phospholipids analysed, with the exception of sphingomyelin in the cold-acclimatized group. Noteworthy is the significant increase in baseline PE in cold-acclimatized animals. (PA, phosphatidic acid; PC, phosphatidylcholine; PI, phosphatidylcholine; PE, phosphatidylethanolamine; PS, phosphatidylserine; SM, sphingomyelin). * indicates P<0.05.
(TIF)
