# Supplementary figures and images for: Cholesterol-Independent Effects of Methyl-β-Cyclodextrin on Chemical Synapses
Source: PLoS One. 2012 May 8;7(5):e36395. doi: 10.1371/journal.pone.0036395 (PMC3348160; doi:10.1371/journal.pone.0036395)

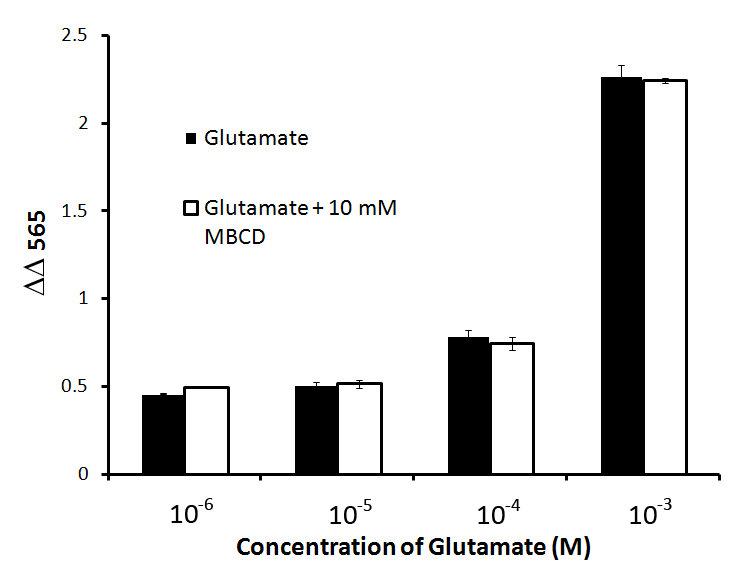

Supplement: Figure S1 — MβCD does not sequester glutamate. Glutamate concentrations were assayed at various concentrations to determine if the reduction in responsiveness to iontophoretically applied glutamate was due to chelation/sequestration by MβCD. Glutamate concentrations were not altered by the presence of MβCD. (TIF) [file pone.0036395.s001.tif]

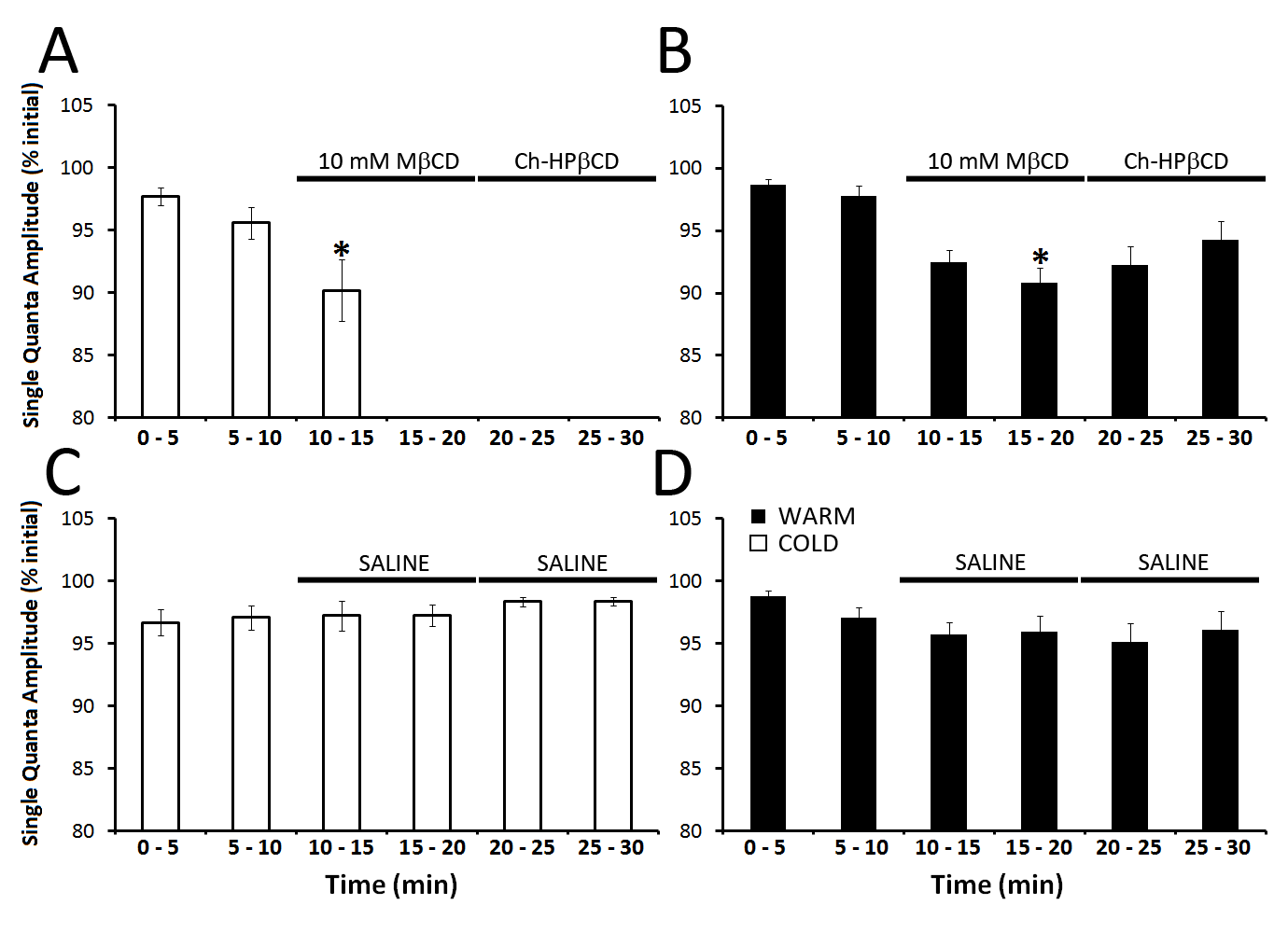

Supplement: Figure S2 — MβCD significantly decreased the amplitude of evoked single quantal events in both acclimatization groups. Extracellular focal recordings were made from nerve branches within abdominal segment IV while stimulating the nerve in segment III. These recordings detected the presynaptic current and postsynaptic response following nerve stimulation. We used the amplitude of single quantal events to determine postsynaptic changes. A. Cold-acclimatized group (open bars). First 10 min reflects saline perfusion. Application of 10 mM MβCD resulted in a significant reduction in the amplitude of single quanta. Since the nerve terminal signal was lost in 6 of 7 trials of the cold-acclimatized group, not enough data could be generated to accurately determine the amplitude of single quantal events after 5 min of MβCD. B. Warm-acclimatized group (closed bars). Application of 10 mM MβCD ultimately resulted in a significant decrease in amplitude of single quanta after 10 min. C-D. Control recordings from the cold- and warm-acclimatized groups, respectively, indicate stable single quantal size over the 40 min recording period. (Stimulation: 0.2 Hz, data were averaged into 5 min bins, giving 60 stimuli per bin. N = 7. * indicates P<0.05.) (TIF) [file pone.0036395.s002.tif]

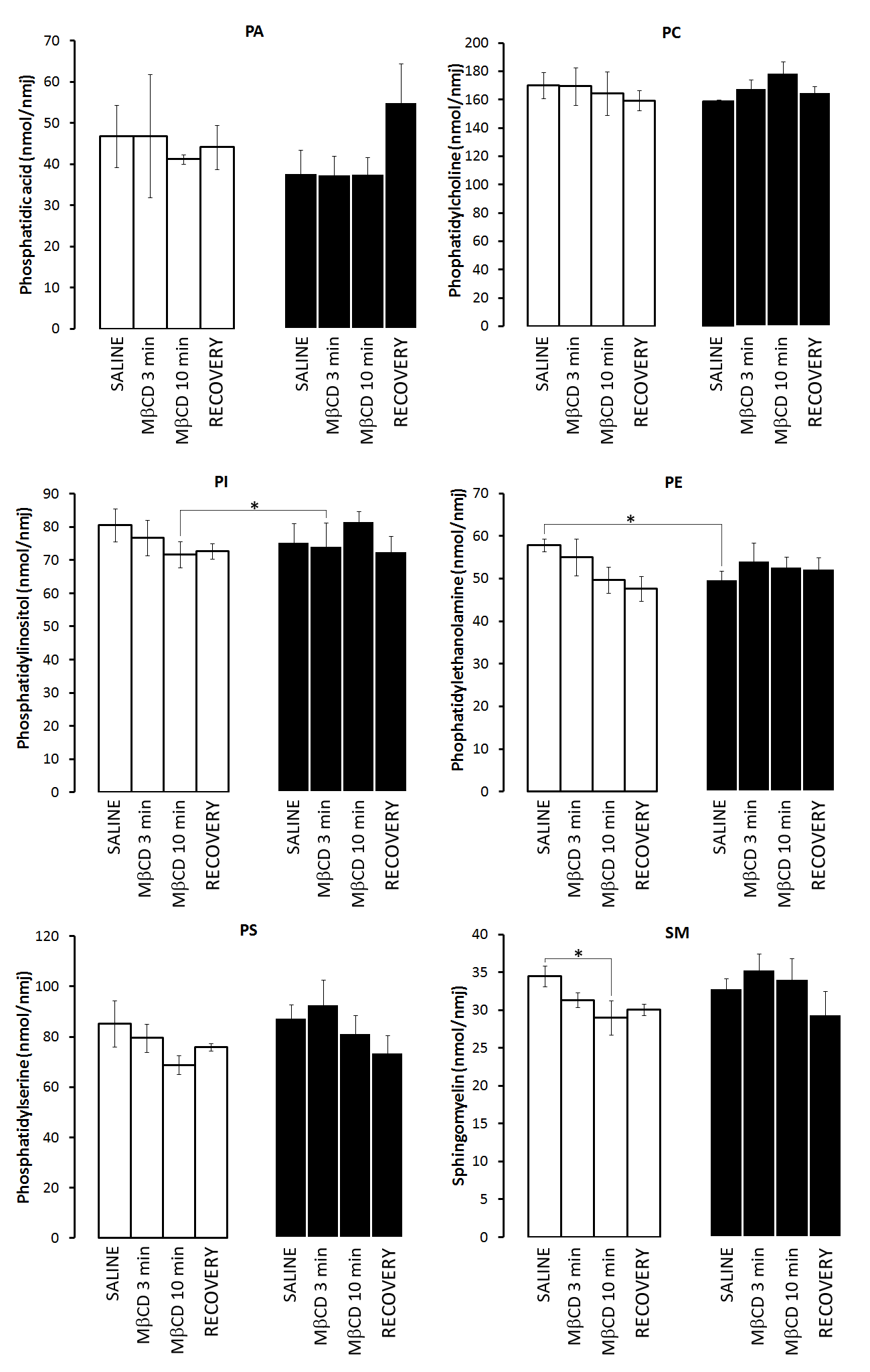

Supplement: Figure S3 — MβCD does not significantly alter the concentrations of other phospholipids in the neuromuscular preparation. Relative concentrations of phospholipids assessed from the same neuromuscular preparation used for the electrophysiological analyses. MβCD did not alter the level of any of the major phospholipids analysed, with the exception of sphingomyelin in the cold-acclimatized group. Noteworthy is the significant increase in baseline PE in cold-acclimatized animals. (PA, phosphatidic acid; PC, phosphatidylcholine; PI, phosphatidylcholine; PE, phosphatidylethanolamine; PS, phosphatidylserine; SM, sphingomyelin). * indicates P<0.05. (TIF) [file pone.0036395.s003.tif]
